# In silico screening of mutational effects on enzyme-proteic inhibitor affinity: a docking-based approach

**DOI:** 10.1186/1472-6807-7-37

**Published:** 2007-06-08

**Authors:** Daniele Dell'Orco, Pier Giuseppe De Benedetti, Francesca Fanelli

**Affiliations:** 1 Department of Chemistry, University of Modena and Reggio Emilia, Italy; 2 Dulbecco Telethon Institute, via Campi 183 41100 Modena, Italy

## Abstract

**Background:**

Molecular recognition between enzymes and proteic inhibitors is crucial for normal functioning of many biological pathways. Mutations in either the enzyme or the inhibitor protein often lead to a modulation of the binding affinity with no major alterations in the 3D structure of the complex.

**Results:**

In this study, a rigid body docking-based approach has been successfully probed in its ability to predict the effects of single and multiple point mutations on the binding energetics in three enzyme-proteic inhibitor systems. The only requirement of the approach is an accurate structural model of the complex between the wild type forms of the interacting proteins, with the assumption that the architecture of the mutated complexes is almost the same as that of the wild type and no major conformational changes occur upon binding. The method was applied to 23 variants of the ribonuclease inhibitor-angiogenin complex, to 15 variants of the barnase-barstar complex, and to 8 variants of the bovine pancreatic trypsin inhibitor-β Trypsin system, leading to thermodynamic and kinetic estimates consistent with in vitro data. Furthermore, simulations with and without explicit water molecules at the protein-protein interface suggested that they should be included in the simulations only when their positions are well defined both in the wild type and in the mutants and they result to be relevant for the modulation of mutational effects on the association process.

**Conclusion:**

The correlative models built in this study allow for predictions of mutational effects on the thermodynamics and kinetics of association of three substantially different systems, and represent important extensions of our computational approach to cases in which it is not possible to estimate the absolute free energies. Moreover, this study is the first example in the literature of an extensive evaluation of the correlative weights of the single components of the ZDOCK score on the thermodynamics and kinetics of binding of protein mutants compared to the native state.

Finally, the results of this study corroborate and extend a previously developed quantitative model for in silico predictions of absolute protein-protein binding affinities spanning a wide range of values, i.e. from -10 up to -21 kcal/mol.

The computational approach is simple and fast and can be used for structure-based design of protein-protein complexes and for in silico screening of mutational effects on protein-protein recognition.

## Background

Among biological macromolecules, enzymes play a crucial role in every cell as catalysts of virtually any biochemical reaction. Kinetics and binding equilibria of enzyme-substrate and enzyme-proteic inhibitor interactions represent the molecular basis of the complex regulatory mechanisms of biochemical pathways.

Enzyme-substrate and enzyme-inhibitor constitute the tightest protein-protein complexes [1], i.e. characterized by very low binding free energies (ΔG°). Comparable high affinities characterize the inter-subunit interactions in some protein quaternary structures (i.e. grow factors, multi-domain proteins etc.) [1].

The ability to modulate the binding affinity in enzyme-proteic inhibitor interactions is of high interest, both for probing the molecular determinants involved in recognition and stabilization of the protein-protein complex, and for unravelling the molecular mechanisms that underlie the early onset of pathological conditions (see for instance Refs. [2,3]). Naturally occurring or artificially induced mutations in either the enzyme or the inhibitor protein represent a convenient way to modulate the binding affinity without altering significantly the three dimensional (3D) structure of the proteins.

Recently, we have developed a rigid-body docking-based approach for estimating the effects of point mutations on the thermodynamics and the kinetics of protein reconstitution [4], and protein-nucleic acid binding [5]. Indeed, we found that, under the condition of an exhaustive sampling of the roto-translational space of one protein with respect to the other, the scoring function (ZD-s) of the ZDOCK2.3 protein docking algorithm [6] has the potential of an empirically determined free energy function for protein-protein and protein-DNA interactions, where no major conformational changes occur upon binding [4]. The fundamental requirement of the approach is an accurate structural model of the complex between the wild type forms of the interacting proteins. The variants (i.e. mutations or deletions) of either one or both the partners can be achieved by molecular modelling. Docking simulations on the wild type forms of the two interacting proteins extracted from the X-ray structure of the complex are bound-bound docking cases. In contrast, docking simulations, in which the modelled mutations concern only one or both the interacting partners, should be moderately assimilated, respectively, to bound-unbound and unbound-unbound docking cases. This is particularly true when mutations involve multiple positions that are essential components of the interface. The basic assumption of the approach is that the architecture of the mutated complexes is almost the same as that of the wild type and no major conformational changes occur upon binding.

**Figure 1:**
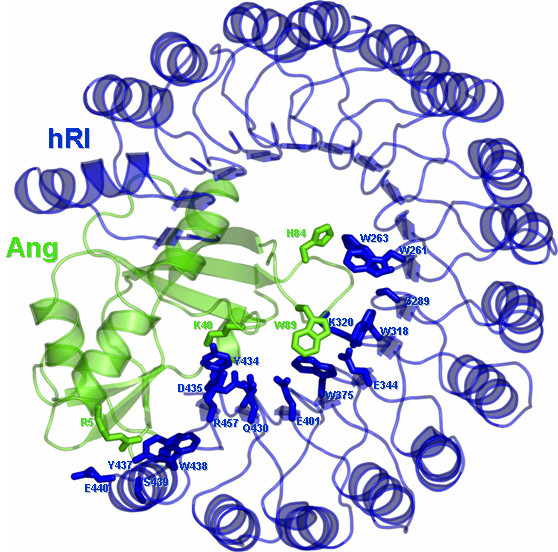
Cartoon representation of the 3D structure of hRI-Ang complex. Residues target of mutagenesis are represented in sticks. Here, as in the following drawings, the protein that was kept fixed in docking simulations (i.e. the target) is coloured in blue, whereas the one sampling the rotational and translational space (i.e. the probe) is coloured in green. Drawings were prepared with the software Pymol [52].

**Figure 2:**
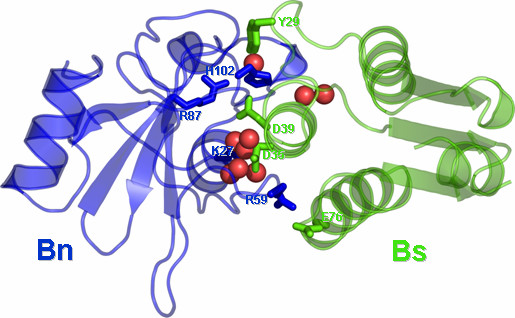
Cartoon representation of the 3D structure of Bn-Bs complex. Residues target of mutagenesis are represented in sticks. Here, as in the following drawings water molecules explicitly included in docking simulations are represented by red spheres.

**Figure 3:**
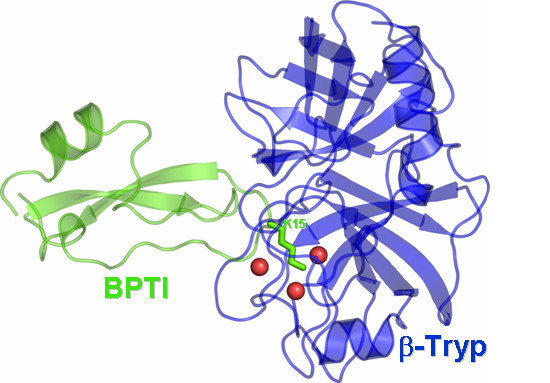
Cartoon representation of the 3D structure of β-Tryp-BPTI complex. Lysine 15 is the residue target of multiple mutagenesis, and is represented in sticks.

In this study, we extend our protocol to three substantially different cases of enzyme-inhibitor recognition, i.e. the human ribonuclease inhibitor-angiogenin (hRI-Ang), the barnase-barstar (Bn-Bs) and the bovine pancreatic trypsin inhibitor-β Trypsin (BPTI-β-Tryp) complexes (Figures 1, 2, 3). The effects of 23 and 15 different modifications (i.e. point mutations or deletions) on the thermodynamics and the kinetics of hRI-Ang and Bn-Bs binding, respectively, have been determined by a number of in vitro experiments (Tables 1, 2, 3) [7-13]. Differently from the hRI-Ang and Bn-Bs systems, in which point mutations are located in either one or both the interacting proteins, in the case of BPTI-β-Tryp, a single amino acid, i.e. K15 in BPTI (K15^BPTI^), was replaced by eight different residues, each substitution exerting a remarkable influence on the binding equilibrium (Table 4).

**Table 1:** Human Ribonuclease Inhibitor (hRI) – Angiogenin (Ang) interaction: thermodynamic and kinetic data and ZD scores

**hRI**	**Ang**	**log *k*_*d*_^a^**	**log *k*_*a*_^b^**	**ΔG°^c ^(kcal/mol)**	**ΔΔG^d^(kcal/mol)**	**N_sol _^e^**	**Best Rank^f^**	**ZD_best_^g^**	**ZD-s^h^**	**ZD_sc_^i^**	**ZD_el_^l^**	**ZD_des_^m^**
Wt^n^	wt	-6.96	8.30	-20.8	-	43	1	82.4	64.2	47.0	17.1	0.1
des (S460)^n,o^	R5A	-3.80	7.72	-15.7	5.1	38	3	74.5	61.0	45.1	14.7	1.2
des (S460)^n^	K40G	-3.14	7.87	-15.0	5.8	38	1	74.8	58.8	44.5	13.5	0.8
Q430A/V432A^n^	wt	-7.08	8.15	-20.8	0.1	45	1	81.7	63.1	46.0	17.1	0.0
W438A/S439A/E440A^n^	wt	-5.77	8.11	-18.9	1.9	45	1	81.8	63.9	45.9	17.3	0.6
R457A^n^	wt	-7.24	8.18	-21.0	-0.2	46	1	82.2	63.6	46.4	17.0	0.2
I459A^n^	wt	-6.70	8.08	-20.2	0.7	47	1	82.0	63.1	46.0	17.3	-0.2
wt^n^	H84A	-6.85	8.28	-20.6	0.2	41	1	80.4	63.9	46.7	17.1	0.1
wt^n^	W89A	-6.80	8.30	-20.6	0.2	44	1	78.7	61.1	44.4	17.4	-0.7
W261A^n^	wt	-7.00	8.20	-20.7	0.1	41	1	81.8	63.2	45.5	17.3	0.4
W263A^n^	wt	-6.16	8.26	-19.7	1.2	41	1	76.0	61.9	45.1	17.4	-0.6
S289A^n^	wt	-7.20	8.04	-20.8	0.0	44	1	82.7	64.4	47.3	17.0	0.1
W318A^n^	wt	-5.89	8.30	-19.3	1.5	37	1	76.6	62.0	45.7	17.4	-1.1
K320A^n^	wt	-7.03	8.46	-21.1	-0.3	45	1	81.9	63.6	46.0	16.8	0.8
E344A^n^	wt	-7.01	8.15	-20.7	0.2	43	1	81.9	63.0	45.9	16.8	0.3
W375A^n^	wt	-6.51	8.00	-19.8	1.0	48	1	77.4	61.8	44.9	17.7	-0.8
E401A^n^	wt	-6.49	8.15	-20.0	0.9	45	1	81.5	63.5	46.1	17.1	0.3
W261A/W263A/W318A^n^	wt	-3.51	7.96	-15.6	5.2	31	11	68.0	57.9	42.5	17.6	-2.2
W261A/W263A/W318A^n^	R5A	-1.39	7.89	-12.6	8.2	28	39	63.7	55.3	40.8	15.3	-0.8
W261A/W263A/W318A^n^	K40G	-0.96	7.59	-11.7	9.2	24	58	61.1	53.9	41.5	14.0	-1.6
Y434A^p^	wt	-5.14	7.88	-17.7	3.1	45	1	78.1	59.8	43.1	17.2	-0.5
D435A^p^	wt	-4.85	8.00	-17.5	3.3	44	1	77.5	59.6	44.6	14.1	0.9
Y437A^p^	wt	-6.51	8.30	-20.2	0.6	41	1	76.8	60.5	43.3	17.5	-0.3
des (S460)^p^	wt	-6.20	8.28	-19.7	1.1	44	1	81.3	63.5	46.0	17.2	0.3

^a^decimal logarithm of the dissociation rate constant *k*_*d*_(s^-1^). Experimental value from Ref. [13].

^b^decimal logarithm of the association rate constant *k*_*a *_(M^-1^s^-1^). Experimental value from Ref. [13].

^c^standard free energy of association as obtained from the relationship ΔG° = RT ln K_D_, where K_D_=*k*_*d*_/*k*_*a*_.

^d^difference in binding free energy for the wild type and variant complexes, calculated from the equation ΔΔG = -RT ln (K_D,*wt*_/K_D,*var*_).

^e^number of native-like solutions among the ensemble of 12000 solutions (i.e. derived from three independent docking runs each ranking 4000 solutions).

^f^rank number of the best native-like solution in the output list (i.e. 1 = highest scored ; 12000 = lowest scored).

^g^ZD scores of the best native-like solution in the output list

^h^ZD scores averaged over all the native-like complexes resulting from three independent runs; ^i^shape complementarity term; ^l ^electrostatic term; ^m ^desolvation term. See *Methods*.

^n^experimental data from [13] (Tables 2, 3, and 5).

^o^recombinant hRI in which Ser460 is deleted. Nomenclature from Ref. [7] ^p ^experimental data from Ref. [7] (Table 4).

**Table 2:** Barnase (BN) – Barstar (BS) interaction: thermodynamic and kinetic data and ZD scores from docking simulations without interfacial water molecules

**BS**	**BN**	**log *k*_*d*_^a^**	**log *k*_*a*_**	**ΔG° (kcal/mol)**	**ΔΔG (kcal/mol)**	**N_sol_**	**Best Rank**	**ZD-s**
wt^b^	wt	-5.43	8.57	-19.00	-	47	1	53.5
wt	H102A	-0.89	8.60	-12.90	6.10	49	1	50.3
Y29A	wt	-3.00	8.46	-15.60	3.40	51	1	49.9
Y29A	H102A	-0.82	8.56	-12.70	6.30	47	1	45.1
Y29F	wt	-5.62	8.48	-19.10	-0.10	47	1	51.3
Y29F	H102A	-1.35	8.59	-13.50	5.50	48	1	47.8
D39A	wt	-0.05	8.28	-11.30	7.70	45	1	48.0
D39A	H102A	1.23	8.64	-10.10	8.90	46	1	46.0
wt	R59A	-2.62	7.53	-13.80	5.20	45	12	44.7
wt	K27A	-2.35	7.71	-13.60	5.40	44	1	51.0
wt	R87A	-1.77	9.33	-13.50	5.50	47	1	51.3
D39A	R59A	n.a.	n.a.	-7.70	11.30	38	32	40.0
D39A	K27A	-0.17	7.76	-10.80	8.20	42	1	46.6
D39A	R87A	-0.52	8.20	-11.90	7.10	46	1	47.7
D35A	wt	-2.42	8.28	-14.50	4.50	48	1	48.9
E76A	wt	-4.68	8.30	-17.70	1.30	52	1	49.7

^a^Details and legend concerning the quantities reported herein were explained in Table 1

^b^Experimental data from [8] (Table 3) and [12] (Table 1)

**Table 3:** Barnase (BN) – Barstar (BS) interaction: thermodynamic and kinetic data and ZD scores from docking simulations with interfacial water molecules

**BS**	**BN**	**log *k*_*d*_^a^**	**log *k*_*a*_**	**ΔG° (kcal/mol)**	**ΔΔG (kcal/mol)**	**N_sol_**	**Best Rank**	**ZD_best_**	**ZD-s**	**ZD_sc_**	**ZD_el_**	**ZD_des_**
wt^b^	wt	-5.43	8.57	-19.00	-	50	1	65.5	55.6	41.6	12.0	2.0
wt	H102A	-0.89	8.60	-12.90	6.10	49	1	63.3	53.0	39.4	11.1	2.6
Y29A	wt	-3.00	8.46	-15.60	3.40	53	1	60.4	52.7	40.1	11.0	1.6
Y29A	H102A	-0.82	8.56	-12.70	6.30	50	1	59.5	50.7	38.1	10.6	2.0
Y29F	wt	-5.62	8.48	-19.10	-0.10	52	1	63.1	54.5	41.8	10.1	2.6
Y29F	H102A	-1.35	8.59	-13.50	5.50	48	1	61.7	51.3	38.6	10.6	2.2
D39A	H102A	1.23	8.64	-10.10	8.90	50	1	60.4	48.6	37.8	6.8	4.0
wt	K27A	-2.35	7.71	-13.60	5.40	48	1	61.5	52.6	40.2	9.7	2.7
D39A	K27A	-0.17	7.76	-10.80	8.20	48	1	59.3	48.3	38.6	5.1	4.5
D35A	wt	-2.42	8.28	-14.50	4.50	50	1	58.2	51.4	38.9	9.7	2.8
E76A	wt	-4.68	8.30	-17.70	1.30	49	1	60.6	53.1	42.2	8.5	2.4

^a^Details and legend concerning the quantities reported herein were explained in Table 1.

^b^Experimental data from [8] (Table 3) and [12] (Table 1)

**Table 4:** β-Trypsin – BPTI interaction: thermodynamic data and ZD scores for X-ray determined and *in silico *modeled BPTI-Lys 15 mutants

**Mutation^a^**	**ΔG° (kcal/mol)^b^**	**N_sol_^xr,c^**	**Best Rank^xr^**	**ZD_best_**	**ZD-s^xr^**	**ZD_sc_^xr^**	**ZD_el_^xr^**	**ZD_des_^xr^**	**ZD-s^xr2,d^**	**N_sol_^xr2^**	**Best Rank^xr2^**	**ZD-s^mod,e^**	**N_sol_^mod^**	**Best Rank^mod^**
GLY	-5.73	63	9	51.7	43.2	34.8	-0.9	9.3	42.3	62	13	42.5	62	1
THR	-7.50	80	1	54.3	45.0	35.8	-1.2	10.4	44.4	79	1	45.5	70	1
ASP	-6.54	76	1	54.9	44.7	37.6	-2.2	9.3	44.8	71	1	44.2	78	1
MET	-10.36	79	1	56.8	47.1	36.7	-1.1	11.5	46.1	78	1	45.4	74	1
GLU	-8.59	73	1	58.7	47.7	38.8	-1.2	10.1	47.3	74	1	44.8	76	1
GLN	-8.73	89	1	55.5	44.8	36.4	-0.8	9.2	45.1	84	1	43.9	74	1
HIS	-9.27	88	1	62.5	48.7	38.6	0.2	9.9	49.4	87	1	47.0	77	1
PHE	-11.04	85	1	67.1	51.8	40.1	-0.1	11.8	51.6	85	1	49.0	71	1

^a^Residue in which BPTI-Lys 15 was mutated. The PDB entries corresponding to these variants are 3BTG, 3BTT, 3BTD, 3BTM, 3BTE, 3BTQ, 3BTH and 3BTF [28]

^b^Standard free energy of association as obtained from the relationship ΔG° = RT ln K_D_. Experimental data from Ref [10]

^c^Here, and in the following columns, *xr *refers to the results of docking runs from the experimentally resolved X-ray structures

^d^Here, and in the following columns, *xr2 *refers to the results of docking runs from the experimentally resolved X-ray structures in the absence of interface water molecules

^e^Here, and in the following columns, *mod *refers to the results of docking runs from the in silico modelled structures of the same complexes

In this study, we reconstituted all the variant forms (Tables 1, 2, 3) of hRI-Ang, Bn-Bs and BPTI-β-Tryp by rigid-body docking, and used this structure-based approach to build a robust quantitative model for determining the residues most relevant for binding, i.e. the so called hot-spots [14].

The results of this study suggest that our computational protocol can be useful for in silico mutational analysis of enzyme-inhibitor interactions, where sufficient structural information on the wild-type forms of the interacting proteins is available.

## Results

### A docking score-based correlative approach for fast estimations of ΔG° in protein-protein interactions

In a recent work we employed rigid body docking simulations for reconstituting nine protein-protein complexes, sharing no structural similarity and characterized by a broad range of ΔG°, i.e from -10 up to -17 kcal/mol [4]. We found that averaging the docking scores of all the native-like solutions from independent docking runs provides an index, i.e. ZD-s, that is linearly correlated with ΔG° [4]. Indeed, we could obtain the empirical equation:

ΔG° = -0.37ZD-s + 2.9 (R = 0.94; p = 0.00015; N = 9)

that was successfully employed to predict the thermodynamics and kinetics of calbindin D9k reconstitution from complementary wild type and mutated fragments [4].

The quality of the correlation and the lack of structural similarity between the training and the test sets led us to the conclusion that this docking-based approach has a general applicability under the condition that no major conformational change occur upon binding. This latter requirement arises form the rigid body approximation and is related to the neglect of conformational and cratic entropy changes upon binding [15].

The cases reported in this study indeed represent an interesting extension of the approach to the important class of enzyme-inhibitor interactions and allowed us to probe the accuracy and the applicability of the method for general protein design purposes.

First of all we probed the ability of the average docking score to discriminate the native-like solutions (i.e. the solutions characterized by a C_α_-Root Mean Square Deviation (Cα-RMSD) lower than 1.0 Å from the native complex) from the false positives. Thus, we subjected to cluster analysis the 4000 solutions provided by each run, by using an algorithm described previously [4]. A Cα-RMSD threshold of 1.5 Å was employed. Benchmarks were carried out on the three protein-protein complexes under study in their native forms, because the crystal structures of such complexes are known. Next we computed the docking scores averaged over all the members of each cluster. Interestingly, for each tested system and for each independent docking run, the cluster characterized by the highest average docking score was indeed that of the native-like solutions. The threshold of 1.5 Å established a clean cut between the cluster of native-like solutions, i.e. the best scored one, and all the other clusters. In general, this happens less frequently with the single docking scores as the highest one does not always correspond to a native-like solution. Hence, the average ZD-s performs better than the single value in distinguishing native-like solutions from false positives.

This was all the more reason for using the average ZD-s instead of the single one in the correlative models (see below in the text).

### hRI-Ang interaction: effects of point mutations and residue deletion on binding affinity

The interactions of hRI with both RnaseA and Ang are well characterized examples of tight protein-protein binding [7,13,16].

The binding affinities of hRI for either RnaseA or Ang are very high, i.e. -18.4 kcal/mol and -20.8 kcal/mol, respectively [7]. In vitro site-directed mutagenesis experiments targeting both hRI and Ang, resulted in changes in the binding affinities, ranging from less than 0.5 kcal/mol for single substitutions up to about 9 kcal/mol for multiple mutations (Table 1) [7,13].

The single or multiple point mutations of hRI and Ang are essentially located at the protein-protein interface, although their positions and features vary remarkably from case to case. Two of the four mutations of Ang, i.e. R5A and K40G, indeed, belong to the interface and reduce the binding affinity because of the breakage of intermolecular salt bridges. In contrast, the remaining two mutants W89A and H84A are less involved in the interface and, consistently, do not substantially affect the binding affinity (Figure 1 and Table 1). As for the hRI mutations, the most detrimental effects on the binding affinity are associated with (a) breakages of H-bonding interactions, like in the case of the Des(S460)^hRI ^deletion, (b) breakages of salt bridges, like in the case of the D435A mutant, or (c) loss of aromatic interactions, like in the cases of the W263A and W318A mutants (Figure 1 and Table 1).

We carried out docking simulations between hRI and Ang in their wild type and 23 variants (Table 1). The native structures of both the enzyme and the inhibitor were extracted from the crystal structure of the complex. In contrast, the variant forms, which include a deletion at the C-terminus of hRI (i.e. Des(S460)^hRI^) and single and triple mutations in either one or both the interacting partners, were built by in silico mutation of the wild type form. The selected docking indices are reported in Table 1 together with the in vitro determined thermodynamic and kinetic data.

The results of docking simulations of hRI-Ang interaction are satisfactory for each tested variant. On average, 41 native-like solutions out of 12000 total solutions from three independent sets of docking runs were found considering all the variants (Table 1). In 20 out of the 24 tested cases, the native-like solutions comprise the best one according to the docking score, i.e. solution N. 1 in the output list (Table 1).

**Figure 4:**
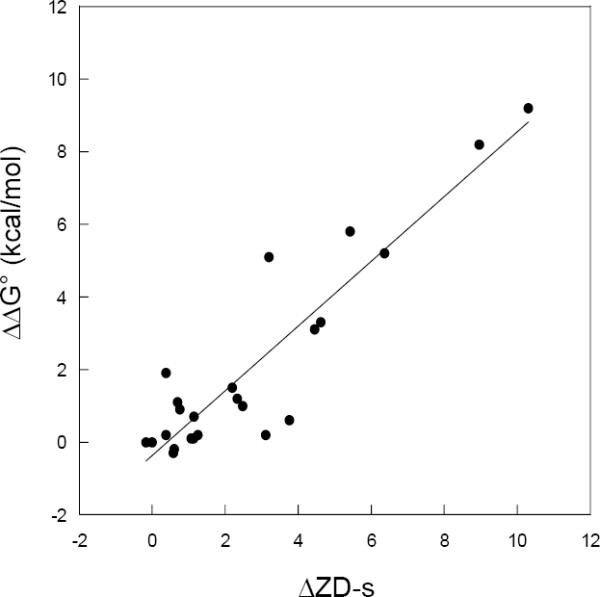
Experimental relative affinities (ΔΔG°) versus relative ZD-s for hRI-Ang interaction. The fitting line equation is ΔΔG° = -0.37 + 0.89ΔZD-s, the correlation coefficient is R = 0.92 and its probability p(R) < 0.0001. The number of experimental points is N = 23.

The correlation between in vitro determined relative binding free energy values (ΔΔG°, see methods) and the relative change of ZD-s for the hRI-Ang systems is reported in Figure 4. The overall trend is clearly linear and the correlation is quantitatively meaningful (R = 0.92, p < 0.0001, N = 23).

In vitro determined ΔΔG° values were also correlated with the relative changes in each of the three components of the relative ZD score, i.e. the shape and electrostatic complementarities (ZD_sc _and ZD_el_, respectively) and desolvation (ZD_des_). All the possible combinations of such components were considered as well (Table 5). The most significant correlations concern that between ΔΔG° and ΔZD_sc _(R = 0.82, p < 0.0001), ΔZD_sc+el_, (R = 0.93, p < 0.0001), or ΔZD_el+des _(R = 0.90, p < 0.0001) (Table 5). These results suggest that combined changes in the shape and electrostatic complementarities are more important contributors to ΔΔG° than the individual terms, and that changes in desolvation do not appreciably affect the thermodynamics of binding.

**Table 5:** Decomposition of ZD-s in three components for the enzyme-proteic inhibitor complexes. Statistical analysis of the empirical correlations

		**ZD_sc_**				**ZD_el_**				**ZD_des_**				**ZD_sc+el_**				**ZD_sc+des_**				**ZD_el+des_**			
	N^a^	A^b^	B^b^	R^c^	p(R)^d^	A	B	R	p(R)	A	B	R	p(R)	A	B	R	p(R)	A	B	R	p(R)	A	B	R	p(R)

HRI-ANG	24	35.5	-0.5	0.82	< 0.0001	10.2	-0.3	0.73	< 0.0001	-2.0	-0.1	0.34	0.105	45.6	-0.9	0.93	< 0.0001	33.4	-0.6	0.73	< 0.0001	8.1	-0.4	0.90	< 0.0001
BN-BS_1^e^	11	56.4	-1.8	0.91	0.0001	-6.4	-0.8	0.56	0.073	-20.3	2.2	0.62	0.042	30.2	-0.9	0.87	0.0005	41.1	-1.3	0.61	0.045	-1.1	-1.1	0.46	0.150
BN-BS_2^f^	11	56.8	-1.8	0.89	0.0002	-9.0	-0.6	0.36	0.282	-19.4	1.7	0.53	0.092	35.5	-1.0	0.81	0.0023	26.3	-1.0	0.50	0.1139	-11	-0.3	0.1	0.7628
BPTI-βtTRYPS	8	31.9	0.9	0.67	0.071	-2.9	0.3	0.57	0.137	6.6	0.6	0.79	0.021	29.0	1.2	0.75	0.032	38.5	1.5	0.82	0.013	3.7	0.9	0.85	0.008

^a^Number of analyzed interactions, i.e. three docking runs for each variant.

^b^Coefficients of the linear regression equation fitting the experimental data. In detail, the equation is in the form Y = A + BX, where Y is the experimental ΔG° expressed in kcal/mol, A (kcal/mol) is the intercept, B (kcal/mol) is the slope and X is the corresponding ZD-s component.

^c^Linear correlation coefficient.

^d^Linear correlation coefficient probability.

^e^Data referring to the protonated form of H102^Bn^.

^f^Data referring to the neutral form of H102^Bn^.

### Bn-Bs interaction: effects of point mutations on binding affinity

The interaction of Bn with its inhibitor Bs is another example of high-affinity protein-protein association (ΔG° = -19 kcal/mol), which has been widely studied in kinetic and thermodynamic detail [8,9,12,17-19].

The importance of electrostatic interactions in driving and stabilizing Bn-Bs association has been widely discussed in the past years [20-26]. In fact, Bs exposes negatively charged amino acids, i.e. D39 and E76, to positively charged amino acids on the Bn surface, i.e. K27, R59 and R87 (Figure 2). Alanine replacement of either one of these amino acids, induces breakages of interfacial salt bridges, therefore diminishing the electrostatic complementarity between the two proteins (Figure 2 and Table 2). In vitro experiments demonstrated that the association rate constant of Bn with Bs is approximately constant over the pH range of 4.5–9, whereas the dissociation rate constant increases by a factor of almost 100 when the pH of the solution is lowered from 7 to 5 [17]. A significant reduction in the pH dependence of the dissociation rate constant of the Bn-Bs complex occurs upon alanine substitution for H102^Bn^[17]. Collectively, these data suggest that the ionization state of H102 does not affect the association process, whereas it influences the dissociation kinetics. The increase in the dissociation rate constant with the lowering of the pH of the solution may be caused by protonation of H102^Bn^. In the crystal structure of the wild type Bn-Bs complex, the Nε nitrogen atom of H102^Bn ^donates an H-bond to D39^Bs^, whereas the Nδ nitrogen atom of the same histidine accepts an H-bond from the backbone amide group of G31^Bs^[27]. This interaction pattern is not compatible with the protonated state of H102 and, consistently, the pK_a _of such histidine in the complex is expected to be lower than 5 [17,24]. Protonation of H102^Bn ^with decreases in the pH of the solution is, therefore, expected to destabilize the interaction pattern found in the Bn-Bs complex, thus favouring dissociation. The structural effects of H102A mutation, thus, include the breakage of two intermolecular H-bonds.

Alanine replacements of R59^Bn^, K27^Bn^, D35^Bs ^also perturb H-bonding interactions with interfacial water molecules. In contrast, loss of van der Waals interactions rather than of H-bonding interactions seems to be responsible for the detrimental effect on the binding free energy exerted by the Y29A^Bs ^mutation. This is suggested by the evidence that the Y29F^Bs ^substitution has a marginal effect on ΔG° (Table 2).

We performed docking simulations between Bn and Bs in their wild type and 15 variants characterized by single mutations on either one or both the interacting proteins, selected for the availability of in vitro determined thermodynamic and kinetic data (Tables 2 and 3). The native structures of both the enzyme and the inhibitor were extracted from the crystal structure of the complex. In contrast, the mutants were achieved by in silico mutating the wild type structure. Consistent with simulations on the hRI-Ang system, docking simulations were in principle carried out without interfacial water molecules. However, since interfacial water molecules are known to be important in Bn-Bs association as they fill regions of poor surface complementarity [27], docking simulations were also carried out by explicitly including water molecules in those cases in which water positions are known at acceptably high resolution (for deep detail, see the Methods section). In particular, these cases include the wild type and 10 Bn and/or Bs mutants (Table 3). Furthermore, both the neutral (i.e. with the hydrogen atom on Nε) and charged forms of H102^Bn ^were considered.

**Figure 5:**
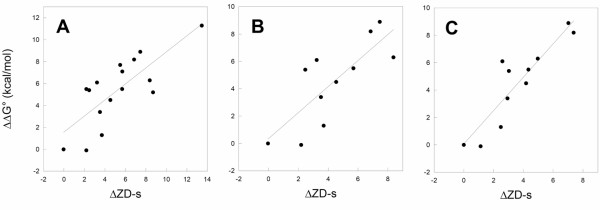
Experimental relative affinities (ΔΔG°) versus relative ZD-s for Bn-Bs interaction. (A) Plot referred to data reported in Table 2, i.e. 16 variants of Bn-Bs without water molecules at the interface and protonated form of H102A^Bn^. The fitting line equation is ΔΔG° = 1.57 + 0.73ΔZD-s, R = 0.77, p(R) = 0.00046, N = 16, where R is the correlation coefficient, p(R) is the probability of such coefficient and N is the number of points. (B) Correlative model derived from the one at point (A) by leaving out four points. The dataset in this plot is limited to the 11 variants of Bn-Bs, for which water positions could be defined at acceptably high resolution (Table 3). The correlation equation and its parameters are: ΔΔG° = 0.35 + 0.95ΔZD-s, R = 0.79, p(R) = 0.0041, N = 11. (C) Same data set as in B, but with ZD-s derived by docking simulations with explicit interfacial water molecules and H102^Bn ^in its protonated state. The correlation equation and its parameters are, respectively: ΔΔG° = 0.06 + 1.22ΔZD-s, R = 0.90, p(R) = 0.00017, N = 11.

Figure 5A reports a plot of the experimental ΔΔG° versus the relative change in ZD-s for the docking simulations without interfacial water molecules and with the protonated form of H102^Bn^. While a trend is clearly observed (R = 0.77, p = 0.00046, N = 16), the correlation is quantitatively less striking than that achieved for the hRI-Ang system (Figure 4). Simulations with the neutral form of H102^Bn ^gave worse correlative models (i.e. R = 0.65, p = 0.006 N = 16).

Comparisons of the correlative models achieved from docking simulations without and with interfacial water molecules for the 11 molecular systems, for which water positions could be defined at acceptably high resolution, showed a remarkable improvement in the correlative models based upon inclusion of explicit water molecules (Figures 5B and 5C). Indeed, the correlation coefficient rises from 0.79 to 0.90 following inclusion of water molecules (Figures 5B and 5C).

Differently from the results of docking simulations without explicit waters, in the case of simulations with explicit waters, the employment of the neutral form of H102^Bn ^does not change substantially the correlative models compared to protonated H102^Bn ^(R = 0.89, N = 11). The ZD-s achieved with protonated and non-protonated H102^Bn ^are, indeed, highly correlated (R = 0.97).

In general, independent of the presence or absence of water molecules and of the prototropic form of H102^Bn ^(i.e. charged or neutral), for each Bn-Bs variant, the best scored solution in the output list (i.e. solution N. 1) was a native-like (Tables 2 and 3).

Decomposition of the ZD-s was carried out only for the cases that produced the best correlative models, i.e. the 11 Bn-Bs variants shown in Table 3, simulated in the presence of explicit water molecules. No significant improvement was observed by using the single components compared to the whole ZD-s (Table 5). More precisely, the improvement on using ΔZD_sc _was marginal (i.e. R = 0.91 with ΔZD_sc _versus R = 0.90 with the whole score). The correlations with ΔΔG° remained acceptable when the ΔZD_sc+el_, is employed (R = 0.87, p = 0.0005), whereas significantly worse linear trends were observed on using desolvation taken either singularly or paired with the other two components (Table 5). The data reported above for the correlative analyses with the ZD-s components refer to docking simulations with protonated H102. The employment of neutral H102 gave very similar results (Table 5).

### Relative binding affinity predictions by merging data from the hRI-Ang and the Bn-Bs systems

**Figure 6:**
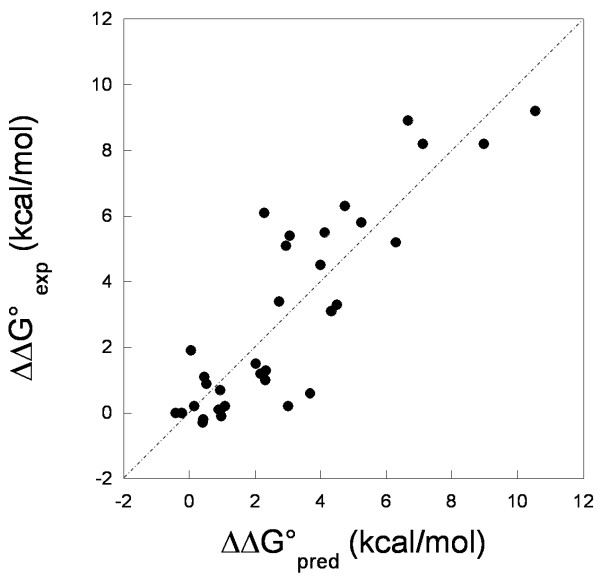
Experimental (ΔΔG°_exp_) versus predicted (ΔΔG°_pred_) relative affinities for hRI-Ang and Bn-Bs data, analyzed as a unique set. The predicted values refer to a leave-one-out test. The fitting line equation is ΔΔG°_exp _= 0.04 + 0.98ΔΔG°_pred_, R = 0.87, p(R) < 0.0001 and N = 34.

As shown in the previous sections, we found convincing correlations between ΔΔG° and the change in the total docking score (ΔZD-s) for both hRI-Ang and Bn-Bs complexes (see Figures 4 and 5). Since such quantity is by definition relative, it was possible to perform a global correlative analysis by merging the two sets of data in a unique set of 34 cases, including 23 data for the hRI-Ang system and 11 data for the Bn-Bs one. The latter concerns only docking simulations with explicit water molecules (i.e. the data set in Table 3). The thereof obtained equation has been used to predict the association free energy changes caused by mutations or deletions in either the enzyme or the inhibitor, or both, by a leave-one-out approach (Figure 6). The trend is significantly linear, the fitting line slope is about 1, and the coefficient R as well as its probability have good values (R = 0.87, p < 0.0001, N = 34).

### β-Trypsin-BPTI interaction: effects of multiple replacements of K15^BPTI^ on the binding equilibrium

The last case considered in this study, i.e. the β-Tryp-BPTI interaction, differs from the other two in that here a single amino acid, i.e. K15^BPTI^, was subjected to eight different substitutions, whose effect on the association equilibrium constant K_A_, i.e. ΔG°, has been determined in vitro (Table 4) [10].

In the wild type structure, the residue target of mutagenesis, K15^BPTI^, protrudes towards a β-Tryp cavity, being highly packed in the binding site (Figure 3). Its functional importance is highlighted also by its central role in coordinating the overall interaction between the two proteins. Indeed, its protonated nitrogen atom is involved in H-bonds with two water molecules and a salt bridge with D189^β-Tryp ^(Figure 3). The latter interaction is considered fundamental for the inhibitory ability of BPTI [28,29].

The heterogeneous physico-chemical nature of the perturbations introduced by the K15^BPTI ^substitutions is reflected by the broad variation of K_A_, i.e. about 4 pK_A _units (Table 4).

Docking simulations were carried out between β-Tryp and eight mutants of K15^BPTI^. Three different molecular systems were considered, characterized by decreasing detail and resolution. The most complete and resolved one consists of the mutants and the relevant interfacial water molecules extracted from the X-ray structures of the complexes with β-Tryp (see Methods for details). The second molecular system, characterized by intermediate resolution, differs from the first one for the lack of interfacial water molecules. Finally, the third and less resolved molecular system is characterized by the lack of interfacial water molecules and by in silico built amino acid substitutions for K15.

As for docking simulations by using the X-ray structures of the K15^BPTI ^mutants and interfacial water molecules, in each case, the best ranked solution (i.e. solution N. 1) is a native-like, except for K15G (Table 4). For each mutant, the average number of native-like solutions out of the total 12000 is about 79.

**Figure 7:**
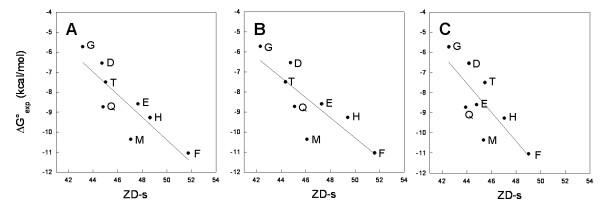
Plot of experimental ΔG° versus ZD-s for the β-Tryp-BPTI complex, where K15^BPTI ^was substituted in eight different amino acids named by their one-letter code. (A) Results of docking simulations starting from the X-ray structures of the eight mutants, including explicit water molecules. The fitting line equation is ΔG° = 17.9 - 0.57ZD-s, R = 0.86, p(R) = 0.006 and N = 8. (B) Correlation model derived by docking simulations on the same molecular models as in (A) but without explicit water molecules. The fitting line equation is ΔG° = 14.8 - 0.50ZD-s, R = 0.82, p(R) = 0.012 and N = 8. (C) Correlation model derived from docking simulations starting from the in silico-modelled structures of the eight mutants, with no interface water molecules. The fitting line equation is ΔG° = 24.2 - 0.72ZD-s, R = 0.79, p(R) = 0.019 and N = 8.

A linear trend was obtained by plotting ΔG° versus the ZD-s (R = 0.86, and p = 0.006, N = 8; Figure 7A). Predicted versus experimental ΔG°'s through a leave-one-out test led to accurate estimations (R = 0.80; p(R) = 0.017, N = 8), which significantly improve if the K15Q substitution is omitted (R = 0.85, p(R) = 0.016, N = 7) [15].

Interestingly, the goodness of the correlative models decreases with the resolution of the structural models (Figure 7). The first slight worsening is observed on neglecting interfacial water molecules in docking simulations (R = 0.86 and R = 0.83 with and without water molecules, respectively, Figures 7A and 7B). A further slight worsening of the correlative models is observed with docking simulations carried out on in silico modelled mutant side chains, neglecting interfacial water molecules (R = 0.79, p = 0.0019, N = 8, Figure 7C). The three linear trends are, however, very similar and share the same outliers, i.e. K15M and K15Q mutants (Figure 7). Although these results support the common knowledge that the performance of the docking algorithms improves with the resolution of the structural models, the results of this comparative study suggest that, at least for the K15^BPTI ^mutants, the performance of the docking algorithm is good even in the case of in silico modelled mutants. It is worth noting, indeed, that, if both K15M and K15Q mutants are deleted, correlations improve significantly for all the three molecular systems, i.e. the ones with highest, intermediate, and lowest resolution (i.e. the correlation coefficients become 0.99, 0.98, and 0.95, respectively). For the K15Q mutant, the side chain conformation of the mutated amino acid is ill-defined even in the crystal structure. Leaving out only this mutant, the correlation for the three systems significantly improves as well (i.e. the correlation coefficients become 0.90, 0.85, and 0.85, respectively).

Dissecting the components of the relative ZD-s derived from the runs on the highest resolved molecular systems shows that the most significant correlations with ΔG° concern ZD_el+des_, (R = 0.85, p = 0.008), ZD_sc+des_, (R = 0.82, 0.013), or ZD_des _(R = 0.79, p = 0.021) (Table 5). Unlike the previous two cases, desolvation seems to play a relevant role in modulating the effects of K15^BPTI ^mutations on the binding free energy.

### Kinetics of enzyme-inhibitor interaction and docking simulations: correlative analysis

Kinetic data (i.e. the association (*k*_*a*_) and the dissociation (*k*_*d*_) rate constants) are available for each variant of the hRI-Ang and Bn-Bs complexes analyzed in the present study (Tables 1 and 3) [12,13]. For the hRI-Ang system, the association rate constants vary about one order of magnitude, whereas, for the Bn-Bs system, they vary two orders of magnitude (Tables 1, 2, 3). Different from the *k*_*a*_*'s*, the *k*_*d*_'s vary to a larger extent, i.e. about six orders of magnitude for hRI-Ang and seven orders of magnitude for Bn-Bs (Tables 1, 2, 3).

For both the hRI-Ang and Bn-Bs systems, changes in the dissociation kinetics, i.e *k*_*d*_, are highly correlated with changes in the equilibrium constant, i.e. K_A _(data not shown).

We searched for empirical correlations between ZD-s and the rate constants, considering also all the combinations of ZD-s components. The results of the analyses are reported in Table 6. For the hRI-Ang system, the most significant correlations with *k*_*a *_were achieved by the total ZD-s (R = 0.71, p = 0.0001, N = 24) and ZD_el+des _(R = 0.76, p < 0.0001, N = 24; Table 6).

**Table 6:** Kinetic parameters for hRI-Ang and Bn-Bs interaction. Statistical analysis of the empirical correlations for the association and dissociation rate constants

**Association (log *k*_*a*_)^a^**																									
		**ZD_sc_**				**ZD_el_**				**ZD_des_**				**ZD_sc+el_**				**ZD_sc+des_**				**ZD_el+des_**			

	**N**	**A**	**B**	**R**	**p(R)**	**A**	**B**	**R**	**p(R)**	**A**	**B**	**R**	**p(R)**	**A**	**B**	**R**	**p(R)**	**A**	**B**	**R**	**p(R)**	**A**	**B**	**R**	**p(R)**

**HRI-ANG**	24	6.3	4.8	0.59	0.002	-16.6	4.1	0.67	0.0003	-6.4	0.7	0.19	0.363	-10.4	8.9	0.74	< 0.0001	-0.1	5.6	0.51	0.0011	-23.0	4.9	0.76	< 0.0001
**BN-BS_1**	11	41.8	-0.2	0.05	0.878	-16.3	3.1	0.50	0.114	12.1	-1.2	0.43	0.188	25.6	2.8	0.32	0.338	53.9	-1.4	0.31	0.343	-4.2	2.0	0.50	0.116
**BN-BS_2**	11	43.0	-0.4	0.08	0.807	-18.7	3.3	0.61	0.045	18.5	-1.9	0.62	0.04	24.3	2.9	0.40	0.225	61.5	-2.2	0.46	0.156	-0.2	1.5	0.44	0.169

**Dissociation (log *k*_*d*_)**^b^																									

**HRI-ANG**	24	40.7	-0.8	0.83	< 0.0001	13.8	-0.5	0.72	< 0.0001	-1.0	-0.2	0.35	0.095	54.4	-1.3	0.93	< 0.0001	39.7	-0.9	0.74	< 0.0001	12.8	-0.7	0.90	< 0.0001
**BN-BS_1**	11	38.3	-0.7	0.93	< 0.0001	8.5	-0.5	0.50	0.116	3.2	0.2	0.57	0.068	46.8	-1.1	0.84	0.0012	41.4	-0.4	0.67	0.025	11.7	-0.2	0.41	0.217
**BN-BS_2**	11	38.4	-0.6	0.91	< 0.0001	8.5	-0.2	0.28	0.410	3.4	0.2	0.45	0.164	46.8	-0.9	0.77	0.0054	41.8	-0.4	0.58	0.063	11.9	-0.02	0.05	0.895

^a^Association process, described by the *k*_*a *_rate constant. The linear regression equation for the overall ZD-s and he *k*_*a *_is, similarly to Table 4, in the form Y = A + BX, where Y is ZD-s, X is log *k*_*a *_and A and B are the coefficients. For hRI-Ang complexes, when the overall ZD-s is correlated with log *k*_*a *_the correlation coefficient and its probability are found to be, respectively, R = 0.71 and p(R) = 0.0001 and the parameters are A = -15.2 and B = 9.5. For Bn-Bs_1 complexes, A = 37.9, B = 1.7, R = 0.25 and p(R) = 0.465, whereas for Bn-Bs_2, A = 43.5, B = 1.0, R = 0.18 and p(R) = 0.593

^b^Dissociation process, described by the *k*_*d *_rate constant. For hRI-Ang complexes, when considering the overall ZD-s, the correlative parameters are A = 53.5, B = -1.5, R = 0.92 and p(R) < 0.0001. For Bn-Bs_1 the correlative parameters are A = 49.9, B = -0.9, R = 0.88 and p(R) = 0.0003, whereas for Bn-Bs_2, A = 50.3, B = -0.7, R = 0.81 and p(R) = 0.002.

In contrast, for the Bn-Bs system, no significant correlation could be found between *k*_*a *_and any combination of the ZD-s components (Table 6).

Interestingly, significant correlations were found between the total ZD-s and *k*_d _for both hRI-Ang (R = 0.92, p < 0.0001, N = 24) and Bn-Bs (R = 0.88, p = 0.0003, N = 11, Table 6). Dissecting the three components of the ZD score shows that, for hRI-Ang, the most significant correlations with *k*_d _are given by ZD_sc+el _(R = 0.93, p < 0.0001) and ZD_el+des _(R = 0.90, p < 0.0001), whereas, for Bn-Bs, the most significant correlations are given by ZD_sc _(R = 0.93, p = 0.0001, N = 11) and ZD_sc+el_(R = 0.84, p = 0.0012, N = 11, Table 6).

The statistic parameters reported above for Bn-Bs concern simulations with protonated H102^Bn^. The employment of neutral H102 gave slightly worsened correlations (Table 6).

### An extended correlative model for absolute binding free energy predictions in protein-protein interactions

**Figure 8:**
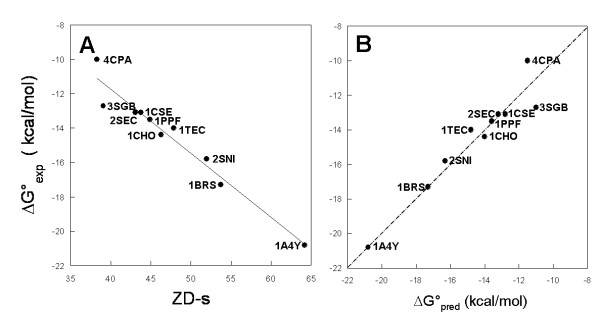
A general quantitative model for docking score-based free energy predictions in protein-protein interactions. (A) Linear correlation between average ZD-s and in vitro-determined standard free energy of association for a set of ten protein-protein complexes. Each dot is labelled according to the PDB code of the complex. Experimental and computational data are reported from Ref [4], except for 1BRS and 1A4Y, which both refer to this study. The linear correlation equation is ΔG° = 3.13 -0.37ZD-s (R = 0.97, p(R)< 0.0001, N = 10). (B) Predicted versus in vitro-determined free energy of association of the same ten complexes. The predicted values refer to a leave-one-out test. The fitting equation is ΔG°_exp _= -0.51 + 0.96ΔG°_pred _(R = 0.96, p(R) < 0.0001, N = 10).

In this study, we reconsidered the quantitative model previously obtained on 9 highly heterogeneous protein systems (see equation (1)), by incorporating the new in vitro and in silico data concerning wild type hRI-Ang. This upgrade extends the binding affinity range by more than 2 kcal/mol (Figure 8 and Ref. [4]). Furthermore, the old ZD-s, concerning docking simulations on wild type Bn-Bs without water molecules, was replaced by the average score from simulations with explicit water molecules. The β-Tryp-BPTI wild type system could not be included in the training set due to ambiguities in the in vitro data [30,31]. The extended equation:

ΔG° = -0.37ZD-s + 3.13 (R = 0.97; p < 0.00015; N = 10)

is strikingly similar to the previous one (equation (1)), i.e. slope and intercept are almost unchanged. This result, considering also that the updated training set covers a wider range of affinities, i.e. from -10 up to -21 kcal/mol, is supportive of the robustness of the quantitative model. The model is, however, more robust in the affinity range from -12 to -17 kcal/mol, Figure 8A). The predictive ability of the extended model was successfully probed by a leave-one-out test concerning the same set of protein-protein complexes (Figure 8B). It is worth noting that the predicted ΔG° for hRI-Ang corresponds to the in vitro value, i.e. -20.8 kcal/mol (Table 1). The same happens for Bn-Bs, for which the predicted ΔG° (i.e. -17.3 kcal/mol) is the same as the in vitro measured value reported by Hartley for the pseudo-wild type C40A^Bn^-C82A^Bs ^(i.e. the form, for which the X-ray structure is available and was employed in this study; see the Methods section) [9]. The value ΔG° = -19 kcal/mol reported in Table 2, indeed, refers to in vitro experiments performed on the wild type Bn-Bs complex.

The model correctly estimates also the binding affinity of the two complementary EF-hand sub-domains that constitute calbindin D9k (i.e. the experimental and predicted ΔG° values are -13.8 and -14.2 and kcal/mol, respectively), by using ZD-s values previously derived by docking simulations of fragment complementation [4].

## Discussion

Recently, we developed a rigid-body docking based approach to estimate mutational effects on the thermodynamics and the kinetics of protein fragment complementation [4] and protein-DNA binding [5]. The approach does not require decomposition of ΔG° in enthalpic and entropic components. The major requirement is, indeed, an accurate structural model of the complex between the wild type forms of the interacting proteins. The variants (i.e. mutations or deletions) of either one or both the partners can be achieved by molecular modelling. The basic assumption is that the architecture of the mutated complexes is almost the same as that of the wild type and no major conformational changes occur upon binding.

In this study, the same approach has been probed in its ability to estimate mutational effects on enzyme-proteic inhibitor binding, focusing on three substantially different systems, i.e. hRI-Ang, Bn-Bs, and BPTI-β-Tryp complexes.

In all the tested cases, the simulated mutations essentially occur at the enzyme-inhibitor interface and their contribution to ΔG° remarkably varies from case to case, depending on whether or not they concern hot-spot regions for the specific interaction. The physico-chemical and geometric features of the interface are quite different in the three considered systems. In fact, a) in the case of the hRI-Ang complex, the interface is mostly made of contacts between aromatic amino acids; b) in the Bn-Bs complex, it is essentially made of ionic interactions; and c) in the BPTI-β-Tryp system, a major contribution from one partner, i.e. BPTI, arises from the amino acid K15, which is involved in charge-reinforced H-bonding interactions (Figures 1, 2, 3). Thus, the extension of the interface is larger for the hRI-Ang and Bn-Bs compared to BPTI-β-Tryp. As a consequence, in the case of BPTI-β-Tryp, computational analyses focused on the functionally important amino acid K15^BPTI^, which was, indeed, subjected to eight different replacements. In contrast, for the hRI-Ang and Bn-Bs systems, mutational analyses involved different amino acid residues on the enzyme, the inhibitor, or both.

Despite the relevant differences in size/shape and electrostatic features of the three considered interfaces, the ZDOCK algorithm was always able to reconstitute the native complex, which was assigned the best docking score in almost every case. Intriguingly, the approach could correctly estimate the effects of point mutations on the binding thermodynamics, hence resulting in good linear trends between experimental ΔΔG° and ΔZD-s (Figures 4, 5, 6, 7).

The relative ZD-s employed in such correlations are simple arithmetic averages over the scores of all the native-like solutions obtained from three independent sets of docking runs. Consistent with previous observations [4,5], the employment of ZDOCK scores averaged over ensembles of native-like solutions performs better in distinguishing native-like solutions from false positives than the ZDOCK scores computed on the best native-like complex. Moreover, the averages perform better than the single score in correlation analyses. In detail, for the hRI-Ang case, the score of the best-ranked solution of each variant (ZD_best_, Table 1) leads to similar correlations with respect to the average (R_ZDbest _= 0.91 versus R_ZD-s _= 0.92), yet with worsened statistical parameters (i.e. standard error on ΔZD-s equal to 0.57, versus 1.20 for ΔZD_best_). As a more general trend, and consistent with previous observations [4,5], ZD_best _performs significantly worse in correlations with ΔG°'s for both Bn-Bs (R_ZDbest _= 0.55 versus R_ZD-s _= 0.90; Table 3) and BPTI-β Tryp (R_ZDbest _= 0.80 versus R_ZD-s _= 0.86; Table 4) interactions. Moreover, the employment of averages overcomes, at least in part, the low resolution of the complexes involving the modelled mutants taking also into account possible slight changes in the binding modes of the mutant structures compared to the wild type. On this line, we have found that the accuracy of predictions is related to the sampling of the native-like solutions; in other words, performance improves with the increase in the number of randomized docking runs and in the consequent number of native-like solutions. Re-docking is, hence, necessary for computing such an average score and is worth doing even in the rare cases, in which the crystal structures are available for both the wild type and the mutated complexes and a simple scoring of such complexes would be possible.

The linear trends remain good when merging the ΔΔG°'s and ΔZD-s's obtained for the hRI-Ang and Bn-Bs systems. The good predictive ability of the quantitative model extended to the two different systems (Figure 6) highlights the potential of our computational approach to handle cases in which it is not possible to estimate the absolute free energies. These correlative models are also suitable for in silico screening of the amino acids relevant to the stability of the enzyme-inhibitor complexes, i.e. hot-spot residues.

Interestingly, our computational approach is effective in handling the peculiar case of the β-Tryp-BPTI system, in which K15, the major contributor from BPTI to the enzyme-inhibitor interface, was replaced by eight physico-chemically different amino acids. In fact, good linear trends were obtained in this case by correlating ΔG° and ΔZD-s, independent of the conformation of the replacing amino acid and of the presence of explicit interfacial water molecules in the simulation. In a very recent study [15], we compared the results obtained with our method for β-Tryp-BPTI with those obtained by applying a well-known empirical approach to K_A _predictions based on changes in the polar/apolar solvent accessible surface area (Δ ASA) [32]. As already demonstrated in a previous study on calbindin D9k reconstitution from complementary fragments [4], the predictive ability of our method, concerning trend and order of magnitude of ΔΔG°, is significantly better compared to the Δ ASA-based method [15].

We have also investigated the role of interfacial water molecules in mediating mutational effects on the binding thermodynamics. We found that, for systems like BPTI-β-Tryp, the presence or absence of explicit water molecules in docking simulations does not affect substantially the linear trends. In contrast, in the case of Bn-Bs, the essential role of interfacial water molecules in mediating protein-protein contacts would require the inclusion of such molecules in the simulations, under the condition that their positions both in the wild type and the mutated complexes are defined.

Collectively, the results of this study suggest that interfacial water molecules should be included in simulations only when their positions are well defined both in the wild type and in the mutants and they result to be relevant for the modulation of mutational effects on the association process.

The correlative analyses by dissecting the ZD-s in the three components, i.e. ZD_sc_, ZD_el _and ZD_des_, also led to interesting results. Collectively, the amelioration of the correlation coefficients on using the single or paired components instead of the whole score did not occurred in all the test cases and, when it occurred, it was marginal. Current and previous results suggest that the whole ZD-s is more suitable for absolute ΔG° estimations concerning heterogeneous molecular systems than the single or differently paired components. However, dissection of the score may provide hints about the physico-chemical determinants of mutational effects on the association process. In detail, for the hRI-Ang and Bn-Bs systems, shape and electrostatic complementarities, considered singularly or in combination, play a major role in modulating the effects of point mutations on the binding free energy compared to desolvation (Table 5). In contrast, in the case of the β-Tryp-BPTI system, desolvation taken alone or paired with each of the other two components plays a major role in modulating mutational effects.

The interpretative and predictive abilities of the correlations concerning the kinetic properties of the enzyme-inhibitor interactions are less striking than those concerning ΔG°. However, in the general context of protein-protein interaction kinetics, the results of this study allow for some interesting remarks. While general rules have been established concerning the diffusion-limited association rates of protein-protein interactions, geometric constraints characterizing the binding sites and physico-chemical processes intervening upon binding cannot be neglected in most of the cases [33]. Indeed, a number of factors influence the kinetics of protein-protein interactions, such as viscosity, ionic strength, pH, and mutations [33,34]. Here, we focus only on the latter contributor. It has been demonstrated that only mutations involving charged residues significantly affect *k*_*a*_, although the magnitude of the effect is strictly related to the specific location of the mutation [33,34]. The most general model of association of two proteins to form a complex is indeed a four-state model including the formation of an unstable encounter-complex and a following intermediate committed to form the final complex [33]. Every step is in principle characterized by specific rate constants, each contributing to the overall rate of association [33]. Simplified models, such as two-state kinetic models, should then be considered approximations that require experimental validation. At best, a rule of thumb for interpreting kinetic data is the notion that short-range interactions mostly affect *k*_*d*_ whereas long-range interactions mostly affect *k*_*a*_. For the systems analyzed in this study, i.e. hRI-Ang and Bn-Bs, the changes in association rate constant upon mutation are of one and two orders of magnitude, respectively (Tables 1, 2, 3). The fact that, for hRI-Ang, *k*_*a *_correlates linearly with ZD_el+des _is in line with the major role played by desolvation and charge-charge interactions in steering the association process. However, despite the relevant role of electrostatics in Bn-Bs association, no significant correlation was found between any combination of ZD-s components and the *k*_*a*_'s for such system, indicative of the low predictive ability of the method concerning mutational effects on the association kinetics. In contrast, the overall ZD-s resulted to be a good predictor of mutational effects on the dissociation kinetics, in line with the high correlation between *k*_*d*_'s and K_A_'s. Collectively, we conclude that our protocol is not suitable for accurately predicting association rate constants, although some interesting insights can be achieved in some cases. A combined approach, including for instance the use of the program PARE, developed by Schreiber and co-workers [35], may eventually lead to a more complete kinetic characterization of the designed complexes.

The extension of equation (1) to equation (2) with the improvement of the statistical parameters, on one hand, and the substantially unchanged fitting curve concerning the ΔG°-ZD-s correlation, on the other, clearly shows that ZD-s, which has now been tested on a broad range of experimental affinities (Figure 8), is, indeed, a good empirical descriptor of protein-protein absolute association free energy. In fact, the developed quantitative model covers a wide range of affinities, i.e. from -10 up to -21 kcal/mol, being, however, more robust in the affinity range from -12 to -17 kcal/mol (Figure 8).

A number of elegant computational approaches have been recently employed to explore the binding energetics and predict mutational effects on the thermodynamics of association of a number of protein-protein systems, including the Bn-Bs and BPTI-β-Trypsin considered in this study [25,36-38]. In this respect, Guerios et al. developed the FOLDEF (i.e. FOLD-X energy function) algorithm for fast estimations of mutational effects on protein stability and protein-protein association [37]. The FOLD-X energy function includes several terms: van der Waals interactions, solvation effects, hydrogen bonds, water bridges, electrostatic and entropic effects for the backbone and side-chains. Backbone entropy is calculated from the secondary structure amino acid preferences derived from the statistical analysis of a protein structure database. Side-chain entropy is calculated by estimated values scaled by a factor that accounts for the loss of side-chain mobility upon burying [37]. Predictions of mutation-induced binding free energy changes were carried out on 82 mutants, whose structural models were obtained by mutating the X-ray structure of the wild type complex. Since the structures of the mutants are identical to that of the wild type except for the mutated amino acid side chain, a necessary condition for the good performance of the approach is that mutation does not induce any structural change in the complex, compared to the wild type. This is a less stringent condition in our approach since the complexes of the mutants are predicted by the docking algorithm and differ, though by a low extent, from that of the wild type. Moreover, as already stated above, the employment of a docking score averaged over the scores of an ensemble of native-like solutions contributes to take into account possible deviation of the mutant complex from the wild type one. The correlation coefficient for the binding free energy changes predicted by FOLDEF versus the calculated ones was 0.64, considering the whole set of 82 mutants [37]. We could compare the predictive ability of our approach with that of the FOLDEF algorithm for the hRI-Ang and Bn-Bs wild type complexes. While our docking-based approach predicts with acceptable accuracy the experimental values for both the hRI-Ang and Bn-Bs ΔG°'s (i.e. -20.8 kcal/mol and -17.3 kcal/mol, respectively (Figure 8B)), the FOLD-X algorithm overestimates the affinity in both cases (i.e. it predicts -31.1 kcal/mol ΔG°, for hRI-Ang, and -21.4 kcal/mol ΔG°, for Bn-Bs). Another approach based on energetics calculation on the X-ray structures of the complexes subjected to global energy optimization of the hydrogen bonding network is that proposed by Kortemme and Baker [36]. The model successfully predicted the results of alanine scanning experiments, i.e. 743 mutations in globular proteins and 233 mutations at 19 protein-protein interfaces [36]. Tested protein-protein complexes included also the Bn-Bs one object of our study. The results by Kortemme and Baker concerning such system corroborate the inference of our study that the explicit inclusion of interfacial water molecules improves the estimation of mutational effects on the thermodynamics of Bn-Bs association. Indeed, the model by Kortemme and Baker neglects water molecules and did not perform so well in mutational analysis of Bn-Bs [36].

Other approaches relied on different molecular simulations protocols [25,38]. In detail, the work by Wang et al. concerned the study of a large set of mutants of the Bn-Bs system by complementary computational methods, in order to estimate the relative importance of different contributions to the binding affinity of the two proteins, to predict mutation-induced ΔΔG°'s, and to conceive a way for designing mutants able to improve Bn-Bs binding compared to wild type [25]. The approach relied on the knowledge of the crystal structure of the wild type complex that was employed to build the structures of the mutants. The minimized structures of the wild type and mutant Bn-Bs were subjected to electrostatic binding free energy calculations by means of the UHBD6.1 program and to Coulombic and Lennard-Jones interaction energy calculations by means of Amber7.0. Decomposition of the Coulombic and Lennard-Jones interaction energies on a per residue pair basis generated 19580 descriptors for each Bn-Bs complex. Multivariate analysis by means of COMBINE PLS led to good predictive models of the quantitative effects of mutations on Bn-Bs binding. The COMBINE analysis model, together with Poisson-Boltzmann electrostatics calculations and Brownian Dynamics simulations gave predictions of the mutants that should bind faster and with higher affinity than the wild type proteins [25]. The QSAR (Quantitative Structure Activity Relationships) model by Wang et al. rely on a multivariate analysis, whereas our correlative models rely on the average docking score taken as a whole or decomposed. Our method takes into account possible differences in the binding mode of the mutants by al larger extent compared to the approach by Wang et al.  Comparisons of the predictive ability of our approach with that of Wang et al. for the set of 11 Bn-Bs complexes (i.e. the wild type and 10 mutants) that gave the best correlative models (Table 2 and Figure 5C) shows a slightly better performance for the COMBINE QSAR model. In fact, the correlation coefficient and the standard deviation concerning the experimental versus calculated ΔΔG by the COMBINE model are 0.95 and 1.13, respectively, whereas those computed through the correlative model reported in Figure 5C are 0.90 and 1.34, respectively.

The work by Almlof et al. concerned an adaptation of the Linear Interaction Energy approach (LIE) to predictions of mutational effects of K15^BPTI ^on the binding to β-trypsin [38]. Where possible, the crystal structures of the wild type and of the mutants were employed. In the absence of the crystal structures, the structural models of the mutants were obtained by mutating K15 in the crystal structure of BPTI. The method, so far limited to single point mutations, consisted of Molecular Dynamics (MD) simulations of the considered mutants and led to accurate predictions [38]. Also in this case, the basic assumption is that mutants bind in the same way as the wild type.

The differences between our approach and that by Almlof et al. are that the former does not require any energy optimization or MD simulations and can handle multiple point mutations with acceptable accuracy. Comparisons of the predicted versus calculated binding ΔG by the LIE method and by the correlative model reported in Figure 7A show the slightly better performance of our model. In fact, the correlation coefficient and the standard deviation are, respectively, 0.75 and 1.62, for the LIE model, and 0.80 and 1.71, for our model.

## Conclusion

Considering the outstanding importance of enzyme activity within cells, computational methods that allow simple and fast predictions of mutational effects on the binding free energy are extremely useful, especially because they may lead to the effective design of specific inhibitors. Whereas a number of first principle-based methods are available today for such purpose, the actual performance is often not balanced by the cost in computer time. So far, our method has been successfully tested on: a) 67 variants of protein-protein associations in water environment (see this work and Ref[4]); b) 32 variants of transmembrane helices dimerization [39]; and c) 10 variants of protein-DNA binding [5], in each case showing consistency with in vitro data.

Our approach is not aimed at investigating the subtle determinants of association for a given protein system, but it is rather aimed at finding the amino acid mutations that cause detrimental effects on the binding energy. The approach is independent of the physico-chemical nature of the protein-protein interface and, since it is based on docking simulations, it can be also applied to cases in which the structure of the complex is unknown even for the wild type.

The method is simple and fast and can be used for structure-based design of protein-protein complexes and for in silico screening of mutational effects on protein-protein recognition.

## Methods

### Rigid-body docking simulations of enzyme-inhibitor interaction and correlation with relative stabilities

Molecular simulations of enzyme-proteic inhibitor interactions were carried out by means of the rigid-body algorithm ZDOCK2.3 [6]. The Fast Fourier Transform-based algorithm performs a search for optimization of molecular complementarity and provides a score (ZD-s) for each docking solution, which can be summarized as a combination of three components:

ZD-s = *α *S_sc _+ *β *S_el _+ *γ *S_des _= ZD_sc _+ ZD_el _+ ZD_des_

where *sc*, *el *and *des *indicate, respectively, the shape complementarity, electrostatic and desolvation terms, and *α *= 0.01*,β *= 0.06, and *γ *= 1 are scaling factors of the energy terms (S) proposed by the ZDOCK developers [40].

When necessary, structural water molecules at the interface were included in the atomic coordinates of the molecule used as a target. The atomic radius and the atomic contact energy (ACE)[41] for the water oxygen atom were thus included in the parameters file, whereas the partial charge was set equal to zero. A 128 × 128 × 128 point grid with a 1.2 Å spacing was used for digitalizing the interacting molecules. As a general rule, the protein with higher molecular weigh was kept fixed (i.e. target), whereas the smaller was allowed to rotate and translate around the target (i.e. probe). A rotational sampling interval of 6° wasemployed, i.e. dense sampling, and the best 4000 solutions were retained for each run and ranked according to the ZDOCK score. Three independent sets of docking runs were performed for each complex, i.e. one starting from the X-ray coordinates and the other two randomizing the initial positions the probe, in a fashion described previously [4]. We selected as native-like structures all the docked complexes characterized by a C_α_-RMSD lower than 1.0 Å from the native complex. This criterion was employed previously [4,5] and demonstrated to be a reasonable threshold. For each docking simulation, both the total score and the three components were averaged over all the native-like complexes resulting from the three independent runs, and employed in the correlation analysis with the thermodynamic and kinetic data. For each tested system, we employed the relative ZD-s index in correlative studies with the relative binding affinity (ΔΔG°). The physical quantity ΔΔG° is defined, for each variant tested in this study, by the relationship:

ΔΔG° = ΔG°_var _- ΔG°_wt_= -RT ln(K_D,wt_/K_D,var_)

where "var" refers to either mutated or deleted forms and K_D _is the equilibrium dissociation constant. All the quantities used in this relationship were taken from in vitro studies (Tables 1, 2, 3 and reference therein).

The CPU time for each docking simulation was found to vary significantly with the size of the docked system. Approximately, each docking run performed on a 2.2 GHz Opteron processor with 2GB RAM took about 23 hours for hRI-Ang (i.e. 3410 and 993 atoms, respectively) and about 8.5 hours for Bn-Bs (i.e. 864 and 693 atoms, respectively) and BPTI-β Tryp (i.e. 427 and 1557 atoms, respectively).

### hRI-Ang interaction: structural information

The structures of both the free and hRI-bound forms of Ang have been solved by X-ray crystallography. The C_α_-RMSD between isolated (PDB entry 1B1I, resolution 1.8 Å) [42] and hRI-bound Ang (PDB entry 1A4Y, resolution 2.0 Å) is 1.07 Å. Reasonably, the highest deviations occur in the 84–90 region, which is involved in the binding interface, and in the 65–69 region, which does not belong to the interface. The overall RMSD is 1.79 Å, indicative of some rearrangements involving the side chains.

The segments named A and B in the 2.0 Å resolution structure corresponding to the PDB entry 1A4Y[11] were used to model the bound structures of hRI and Ang, respectively. Table 1 shows the single or multiple point mutations performed in either hRI or Ang in each docking run. Mutations were performed by means of the Protein Design module within the QUANTA2005 package [43]. For each docking simulation, hRI was used as a target while Ang was used as a probe.

### Bn-Bs interaction: structural information

The structures of the Bn-Bs complex and of the isolated components have been determined at the atomic detail. The C_α_-RMSD between unbound (PDB entry 1A2P, resolution: 1.5 Å) [44] and Bs-bound Bn (PDB entry 1BRS, resolution: 2.0 Å)[27] is 0.46 Å, whereas the overall RMSD equals 0.77 Å. In contrast, the Cα- and the overall RMSDs between unbound (PDB entry 1BTA, average NMR structure)[45] and Bn-bound Bs (PDB entry 1BRS, resolution: 2.0 Å) are 0.87 Å and 1.41 Å, respectively.

The Bn and Bs structures extracted from the 2.0 Å resolution complex (PDB entry 1BRS [27]) were employed in docking simulations. Three complexes are present in the crystallographic unit, which slightly differ in completeness and few side chain assignments. Quality checks through the Protein Health module of QUANTA2005 led to the final choice of the complex corresponding to the segment A for Bn (residues 3–110) and D for Bs (residues 1–89). In case of multiple side chain assignment, i.e. that of S14^Bs^, the criterion was to choose the conformation improving the stereochemical quality of the structural model. Tables 2 and 3 report the mutations performed in either Bn or Bs in each docking run. Mutations were performed by means of the Protein Design module within the QUANTA2005 package, and the Ponder's rotamer library [46] was applied to assign the conformation to the Y29F substitution. The amino acid residue H102 from Bn was considered both in its neutral (i.e. with the hydrogen atom on Nε) and charged forms. Docking runs on the wild type and mutated forms of Bn and Bs were performed both in the absence and in the presence of structural water molecules. Simulations without explicit water were carried out on 16 molecular systems (i.e. the wild type and 15 mutants (Table 2). In contrast, simulations with explicit water concerned a more limited set of variants (i.e. the wild type and 10 mutants, Table 3), for which information on the positions of interfacial water molecules could be obtained from the X-ray structures. In detail, the included water molecules are numbered as 14, 22, 29, 33, 36, 48, 93, 128 and 155 in 1BRS, which mediate Bn-Bs contacts and show an almost fixed position in all the X-ray structures of the complexes resolved so far, i.e. that of the wild type (1BRS [27]) and those of the mutants K27A^Bn^/D35A^Bs ^(1B2U [47]), H102A^Bn^/Y29F^Bs ^(1B3S [47]), K27A^Bn^/T42A^Bs ^(1B2S [47]), E76A^Bs ^(1X1X [48]) and Q2A/D35A^Bs ^(1X1Y [48]). Only for the mutant H102A^Bn^/Y29F^Bs^, interfacial water molecules 22, 33, 93 and 128 (the numbering is that from the 1BRS structure) have been omitted because they are not present in the crystal structure of the complex (1B3S [47]). The inclusion of these water molecules was done because of their known importance in improving the shape complementarity between Bn and Bs. In addition to the interfacial water molecules common to the wild type and the mutants, ad hoc water molecules that occupy the cavity formed upon alanine replacements were included in the modelled structures of selected mutants. In detail, (a) the water molecule 48 (i.e. wat48), extracted from the structure of the K27A^Bn^/D35A^Bs ^mutant (1B2U), was included in the K27A^Bn ^mutant, whereas wat2 and wat35, extracted from the same crystal structure, were included in the D35A^Bs ^mutant; (b) wat58 and wat98, extracted from the structure of the H102A^Bn^/Y29F^Bs ^mutant (1B3S), were included in the H102A^Bn ^mutant, whereas wat65 and wat85, extracted from the same crystal structure, were included in the Y29F^Bs ^and Y29A^Bs ^mutants; finally (c) wat152 and wat211, extracted from the crystal structure of the E76A^Bs ^(1X1X [48]) mutant, were included in the E76A^Bs ^mutant. The mutants D39A^Bs^, R87A^Bn^/D39A^Bs^, R59A^Bs^/D39A^Bn ^and R87A^Bn ^were excluded from calculations with explicit water because information on ad hoc water molecules that would fill the cavity left by mutation is lacking. We decided to consider D39A^Bs ^only in docking simulations with the K27A and H102A Bn mutants, for which information on the positions of ad hoc water molecules is available (see above). For each mutant, water molecules were considered as belonging to Bn, which was kept fixed (i.e. target), whereas Bs was chosen as a probe.

It is worth noting that, in this work, the "wild type Bn-Bs" indeed refers to the complex holding the C40A/C82A double Bs mutant, elsewhere named as "pseudo-wild type" [47]. This form of Bs shows a slightly lower affinity for Bn compared to the wild type (i.e., ΔG° = -17.3 kcal/mol)[9] and the high resolution structure in complex with Bn is indeed available only for such mutant [27]. NMR relaxation spectroscopy, however, clearly showed that the structure of barstar C40A/C82A is essentially the same as that of the wild type [49], hence validating the use of the pseudo-wild type as a template in our study.

### BPTI-β-Tryp interaction: structural information

Smith et al. evaluated the total interface RMSD between the bound and unbound forms of BPTI interacting with β-Tryp to be, respectively, 0.57 Å for β-Tryp and 0.98 Å for BPTI [50].

From X-ray crystallography, two 3D-structures of the wild type BPTI-β- trypsin complex are available at 1.85 Å (PDB entry 2PTC)[51] and 1.90 Å resolution (PDB entry 3BTK) [28]. Moreover, the structures of the complexes between ten mutants of the BPTI residue K15 and β- Tryp are available at an average resolution of 1.90 Å (PDB entry 3BTX, where X indicates the code of the amino acid substituting Lys) [28]. Two of these structures, i.e. 3BTK and 3BTW, were excluded from the correlative analysis because the relative in vitro data were not homogeneous with respect to the data concerning the other K15 mutants. In detail, the thermodynamic data concerning the wild type form, i.e. 3BTK, refer to an early work [31] and were assessed in different conditions than those of all the mutants [10]. On the other hand, the K15W mutant, 3BTW, presents high B factors of several side chains in the binding pocket as well as two less clearly defined water molecules [28], which make it a more ambiguous case if compared to other mutants. Docking simulations of the eight variants of BPTI, i.e. the probe, were run both by including and excluding structural water molecules at the interface as bound to β-Tryp, i.e. the target. Where ambiguous side chains assignments were present, alternative conformations were tested (data not shown). Moreover, the same eight BPTI mutants of known 3D structure were built by introducing the point mutation in the structure of the wild type protein, and then used as probes in docking simulations. In silico mutations were carried out on the 2PTC structure, characterized by better stereochemical quality compared to the 3BTK structure. The Ponder's rotamer library [46] was employed to assign the conformation to the mutated amino acids. No water molecules were added at the interface in order to avoid any possible bias arising from experimental information.

## Authors' contributions

DDO conceived the present study, carried out docking simulations and analyses. PGDB contributed to supervise the work. FF initiated the project and supervised the work. DDO and FF shared the writing of the manuscript. All authors read and approved the final manuscript.
